# The ANZAED Eating Disorder Credential for health care providers: clinician perspectives

**DOI:** 10.1186/s40337-025-01309-8

**Published:** 2025-06-16

**Authors:** Katarina Prnjak, Janet Conti, Madalyn McCormack, Gabriella Heruc, Siân A. McLean, Rebecca Barns, Phillipa Hay

**Affiliations:** 1 Graduate School of Health, University of Technology Sydney, Sydney, Australia; 2 School of Psychology, Western Sydney University, Sydney, Australia; 3 Translational Health Research Institute, School of Medicine, Western Sydney University, Sydney, Australia; 4 Eating Disorders and Nutrition Research Group, Translational Health Research Institute, School of Medicine, Western Sydney University, Sydney, Australia; 5 School of Psychology and Public Health, La Trobe University, Melbourne, Australia; 6 Mental Health Services, Campbelltown Hospital, Campbelltown, Australia

**Keywords:** Anorexia nervosa, Bulimia nervosa, Binge eating disorder, Continuing professional development, Clinician supervision

## Abstract

**Background:**

Australia & New Zealand Academy for Eating Disorders (ANZAED) in 2022 established a credentialing system for eating disorder (ED) clinicians that recognises a minimum knowledge, training, and ongoing professional development necessary to provide safe and effective care. The aim of this study was to explore experiences of credentialed clinicians with the new credentialing system, in particular, their view on continuous professional development (CPD) and supervision required for maintaining the credentialed status, as well as how becoming credentialed has affected their clinical work.

**Methods:**

Two hundred and twenty-eight credentialed clinicians (92.5% female; 41.7% psychologists; 39.5% dietitians) completed an online survey consisting of multiple choice and open-ended questions regarding their experience with the credentialing system and perceptions of the CPD program.

**Results:**

Credentialed clinicians reported that CPD increased their confidence and willingness to deliver ED treatment, and that supervision enhanced their reflective skills and ethical thinking, whilst 75% of clinicians reported that attaining the Credential had not changed the number of ED patients that they were currently treating on a weekly basis. Content analysis of open-ended questions resulted in three broad themes: professional/personal development; improving care; and acknowledgement and recognition. Differences were found between clinicians working in private practice and those working in public health or both settings with regards to impacts on client referrals to their services.

**Conclusions:**

These findings show that credentialed clinicians perceived a positive experience with the Credential and its ongoing CPD program. However, there may be a need to increase the visibility and external awareness of the credentialing system.

## Introduction

Eating disorders (EDs) are amongst the most complex conditions to treat [1], with only half of individuals diagnosed with an ED reaching full recovery [2]. Reasons cited for this have included a scarcity of qualified treatment providers and beliefs held by clinicians about adopting and implementing evidence-based treatment [3]– although not everyone is responsive to evidence-based treatment as reflected in results of randomised controlled trials, which is why moving towards more personalised medicine in treatment of EDs is also needed [4]. Access to treatment is also relevant. For example, a systematic review by Johns and colleagues [5] found that lack of knowledge and understanding as well as experience dealing with EDs among primary care professionals (GPs, nurses, social workers) were reported by lived experience individuals as a barrier towards treatment. They also believed that primary care professionals require more time and resources to diagnose and treat EDs, as well as more interest in understanding this type of psychopathology. Moreover, carers of individuals with EDs reported seeking help online, in self-help books, and support-based organisations other than primary care or community settings [5]. Carers have described the process of help-seeking as a “long arduous journey” [6] and talked about knowledge of available treatment options as well as challenges in treatment access across a range of healthcare systems. Likewise, professionals such as dietitians and GPs have also reported feeling untrained to detect and manage ED presentations [5, 7, 8]. Equipping health professionals with specific knowledge and skills thus may prevent individuals with EDs falling through the cracks of care.

Credentialing programs may overcome some of these factors impeding access to ED treatment and timely recovery. For example, the International Association of ED Professionals (IAEDP) has a certification program for ED professionals [9, 10]. It however has not been taken up in Australia and has not been consistently endorsed by other international professional organizations [10]. It has a large emphasis on knowledge testing and less emphasis on professional development, and to the best of our knowledge its effects haven’t been empirically evaluated. The Australia & New Zealand Academy for Eating Disorders (ANZAED) established a credentialing system that in contrast has a large emphasis on professional development and recognises ED clinicians with a minimum knowledge, training, and ongoing professional development necessary to provide safe and effective care [11, 12]. The criteria for the Credential were built on the National Eating Disorders Collaboration (NEDC) Workforce Core Competencies [13] and the ANZAED Clinical Practice & Training Standards [14]. The ANZAED Eating Disorder Credential was launched to the public in June 2022, with 1369 clinicians joining up to May 2023.To maintain their credentialed status each year, clinicians are required to complete 6 h of supervision relevant to EDs as well as 15 h of Continual Professional Development (CPD) relevant to EDs. For more details on the ANZAED Eating Disorder Credential please refer to the credentialing website (www.connected.anzaed.org.au).

Credentialing systems have been implemented more broadly among several professionals in the US, such as nurses [15], applied behavioral analysts [16], and psychologists working in a hospital setting [17] with benefits and drawbacks identified. For instance, in public health nursing, credentialing was shown to have a high personal value, yet credentialed professionals argued that more effort is needed to enhance its external recognition, and lack of financial benefits to the credentialed clinicians was highlighted as the greatest barrier towards becoming credentialed [18]. Similar findings were reported by Vandenhouten et al., [19] who highlighted the lack of awareness of the credential and its eligibility criteria (as well as large number of practice hours required) as the main barrier to becoming credentialed, with additional barriers being perceived lack of value by employers and the time needed to prepare for the exam. On the other hand, Simmons and colleagues [20] found that credentialed dietitians perceived improvements in their collaborative practice after being credentialed, such as experiencing more comfort communicating with the other members of the healthcare team, feeling their clinical decisions were cost-effective for clients, feeling like a valued member of the healthcare team, and observing that their recommendations were often implemented by the healthcare team relative to before becoming credentialed. The area of the least improvement as a result of becoming credentialed was increase in salary, with only 19% of study participants reporting increased salary post-credential [20]. Credentialed professionals across these studies thus appear to be motivated by factors related to professional competence [19] but other, more personal, factors could also play a role.

It is also possible that processes or requirements that are embedded within credentialing initiatives, such as training or professional development specifications, confer benefits to clinicians. A recent study of Australian dietitians working in the ED field evaluated newly implemented peer supervision and reported participants experienced increased confidence to provide evidence-based care, improved reflective practice, heightened feelings of enjoyment as well as alleviated stress associated with the ED workload [21]. Almost all (98%) of participants stated that the supervision positively affected their clinical practice although individual rather than peer group supervision was slightly preferred by the dietitians as the main mode of clinical upskilling [22]. These findings suggest that the ongoing requirements of a credentialing system can support the continuing development of practice.

Given the scarcity of research and mixed findings regarding credentialing systems for ED health care professionals, the aim of this study was to explore the experiences of the credentialing process, effects that becoming credentialed may have on their clinical practice, as well as perspectives on supervision and CPD requirements necessary for maintaining the Credential, as perceived by clinicians awarded the ANZAED Eating Disorder Credential. In addition, we sought to understand which clinician-related factors would contribute to perceptions of the experience and value of the Credential. Given the exploratory nature of this study, and lack of research into credentialing within the ED field, we did not pose specific hypotheses.

## Methods

### Design and procedure

This study used a cross-sectional quantitative survey design. The study was approved by the Western Sydney University Human Research Ethics Committee (HREC Approval Number: H15252). All participants gave informed consent. Participation was voluntary and participants could withdraw from the study at any time. A purposive sampling method was employed to recruit clinicians awarded the ANZAED Eating Disorder Credential in Australia. Specifically, Credentialed Eating Disorder Clinicians were identified and contacted directly via their listed email address on the Credential’s clinician database (connect·ed; *n* = 800). The data were collected via an online survey using the Qualtrics platform. Data collection commenced February 2023 and was completed March 2023. Reminder emails were sent over two waves. Potential participants were sent an email invitation with a hyperlink to the Qualtrics site, in which they could complete the questionnaire. The invitation provided information about the purpose of the study, anticipated completion time, and compensation for participation. At the beginning of the survey, individuals provided informed consent in order to proceed with the study. Participants could withdraw from the study at any time, without consequences.

Clinicians were only included if they consented to the study and confirmed that they were a Credentialed Eating Disorder Clinician and over 18 years old. If individuals did not provide this information, then they were unable to continue with the study. To keep data confidential, participants were asked to generate a unique ID code. Most closed questions were forced choice to minimise missing data. The survey took approximately 15–25 min to complete. Participants were reimbursed for completing the survey with a $50AUD gift voucher, and an additional $50AUD gift voucher for participating in the interview.

### Measures

The online survey, with a total of 81 items, consisted of the following six parts: (i) demographics; (ii) profession (e.g. job title) and ED treatment experience (e.g. years worked in the ED field, age groups and ED presentations worked with); (iii) the ANZAED Eating Disorder Credential (e.g. views on the Credential, impact of the Credential on number of clients seen); (iv) finding treatment providers (e.g. using the connect·ed website to refer clients); (v) supervision (e.g. experience as a supervisee as well as, if relevant, experience as a supervisor); and (vi) CPD (e.g. perceptions of ED-related CPD as providing greater understanding about the topic). Overall, 78 of the questions were closed and three questions were open-ended. Questions around supervision were rank ordered on a Likert-type scale where 0 was “strongly disagree” and 5 was “strongly agree.”

### Data analysis

A statistical power analysis was conducted on G*Power 3.1 desktop software using a medium effect size (f = 0.25). It was revealed that a sample size of 72 participants (alpha = 0.05 and power = 0.90) was required to obtain a statistically significant R2 for up to 5 tested explanatory variables in the regression analyses. Thus, the proposed sample is adequately powered to detect these effects.

Quantitative analyses were conducted using SPSS (Version 24.0). Linear regression analyses were conducted with profession (mental health vs. non-mental health), years practising, work sector (public, private, or both), geographical setting (metropolitan, regional, rural, remote) and number of clients with an ED as predictor variables. Two separate analyses were conducted with “overall experience of the Credential” and “perceived value of the Credential” as outcome variables. Gender, age and ethnicity were controlled for in these analyses.

Content analysis of qualitative data was conducted using NVivo software version 14 (2023). Responses to two open-ended questions were analysed: “Please share with us your views in general on the credentialing of clinicians?” (*n* = 225) and “Please share with us what motivated you to become a Credentialed Eating Disorder Clinician?” (*n* = 226). Two authors separately read participants’ responses, extracted codes, and proposed themes. Then they met several times to discuss their views and to reach a consensus with the help of a third author. Once themes and subthemes were finalised, we inspected their frequencies across participant attributes (case classifications).

#### Reflexivity

KP is a caucasian cis-gender woman and a provisional psychologist with 7 years of experience conducting research in the field of EDs. MM is a caucasian cis-gender woman, psychologist, and a Credentialed Eating Disorder Clinician with clinical experience working with adults with EDs. PH is a caucasian cis-gender experienced female Psychiatrist, Credentialed Eating Disorder Clinician, and researcher in the field with a lived experience of ED. SM is a caucasian cis-gender woman, a researcher in the field of EDs, and a contributor to the development of the ANZAED Eating Disorder Credential. GH is a caucasian cis-gender experienced female Accredited Practising Dietitian, Credentialed Eating Disorder Clinician, researcher in the field of EDs and the Credentialing Director for ANZAED. JC is a caucasian cis-gender experienced female Clinical Psychologist, Credentialed Eating Disorder Clinician and qualitative researcher. RB is a caucasian cis-gender woman and a clinical psychologist with 10 years of experience working with people with EDs and over 3 years experience working in the research field with people with EDs.

## Results

### Participant characteristics

The survey collected data from 228 Credentialed Eating Disorder Clinicians on their experience providing treatment. Most clinicians in the sample were female (92.5%) with the mean age of 38.3 (range: 24–72; SD = 10.0). More than half (58.8%) of the clinicians surveyed reported that their cultural and ethnic background was Oceanian and 1.3% of clinicians identified as being of Aboriginal origin.

Of the credentialed clinicians who participated in this study, 41.7% were psychologists, 39.5% dietitians, 18.4% other mental health professionals, and less than 1% medical practitioners. All participants resided in Australia, with most clinicians reporting that they practiced in New South Wales (32.9%), Queensland (25.4%), or Victoria (20.6%). This distribution is similar to the distribution of credentialed clinicians listed on the connect·ed website. Around one third of clinicians reported working primarily in regional or rural/remote locations. Most spoke English at work (97.8%). Participants had a median of 5 years (IQR: 2–9) of experience providing care for people experiencing EDs and just over half of the clinicians (51.8%) indicated that over 50% of their clients have had an ED. The most common ED presentation seen by participants was Anorexia Nervosa (AN; 77.6%). Most clinicians provided treatment in the outpatient setting (93%) with majority offering an in person appointment (92%). The most widely used treatment modality was individual therapy (41.2%) or a combination of individual and family therapy (42.1%), while providing group therapy (< 1%) or family therapy alone (1.8%) was relatively uncommon. Most clinicians (75%) reported having seen a substantial proportion (> 25%) of people with EDs in their clinical practice within the last year, and 62% of clinicians spent two or more days a week working with people with an ED. For further details about the participants, see Table 1.

**Table 1:** Descriptive statistics of credentialed clinicians who participated in this study

Clinician characteristic (*N* = 228)	*Descriptive statistic*	
	*n*	Percent (%)
Ethnicity*		
Oceanic	134	58.8%
European	45	19.7%
Asian	10	4.4%
Other/prefer not to say	12	17.1%
**State/Territory of residence**		
New South Wales	75	32.9%
Queensland	58	25.4%
Victoria	47	20.6%
Western Australia	23	10.1%
Tasmania	10	4.4%
Other	15	6.6%
**Geographical area of practice**		
Metro	147	64.5%
Regional	54	23.7%
Rural/Remote	11	4.8%
Mixed	16	7%
**Practice sector**		
Private sector	120	52.6%
Public health	55	24.1%
Public and private sector	53	23.2%
**Service setting**		
Eating disorder specific	85	37.3%
General mental health	91	39.9%
General health	52	22.8%
**Age groups providing services for**		
Adult only (18–64 years)	44	19.3%
Child to adult (< 65 years)	40	17.5%
Youth and adults (16–64 years)	37	16.2%
Any age	34	14.9%
Child and/or youth (up to 25 years)	28	12.3%
Child and adolescent only (0–18 years)	23	10.1%
Youth to older persons (16–65 + years)	22	9.6%
Therapy modality*		
Individual	94	41.2%
Individual and Family	96	42.1%
Group with/without family/individual	31	13.6%
**Type of eating disorder commonly treated**		
Anorexia Nervosa	177	77.6%
Atypical Anorexia Nervosa	174	76.3%
Binge Eating Disorder	147	64.5%
Bulimia Nervosa	135	59.2%
Avoidant/Restrictive Food Intake Disorder	78	34.2%
Binge Eating Disorder (subthreshold)	56	24.1%
Bulimia Nervosa (subthreshold)	48	21.1%

Note. Response categories were not mutually exclusive, so percentages do not add up to a 100. *Some survey items were not set up to force a response, and some participants chose not to respond. For these survey responses the total number of responses is less than 228

### Perceptions of the credential and its processes and requirements

Three quarters (75.4%) of clinicians reported that attaining the Credential had not changed the number of patients with an ED that they were currently treating on a weekly basis. Of the 24.6% of clinicians who reported that their caseload had changed, 47 clinicians predicted that on average they would be able to provide treatment to 20 (SD = 20.2) more individuals with an ED within the next 12 months.

Table 2 presents clinician responses regarding their perceptions of the Credential value, processes, and CPD requirements. Importantly, 80.7% and 69.5% of clinicians somewhat or strongly agreed that CPD increased their confidence and willingness to deliver ED treatment, respectively. In relation to supervision, the vast majority of clinicians (96.5%) reported receiving supervision, of which 52.8% were engaged in interdisciplinary supervision and 37% providing supervision to other clinicians. ED-specific supervision was perceived as a safe space to reflect on practice by 88.9% of participants, whilst 78.5% and 75.1% reported that receiving supervision enhanced their reflective skills and ethical thinking, respectively. Furthermore, 86.9%, 84.0% and 73.3% agreed that receiving supervision increased their confidence, ability and willingness to deliver interventions, respectively. Finally, 70.8% of clinicians believed that the ongoing supervisory requirements for maintaining their Credential status were reasonable.

**Table 2:** Participant responses to questions about the credentialing system

Survey questions and response options	Response distribution (%)
How highly you value the Credential for your individual professional practice?	
*not at all*	0
*only a little*	10.0
*to some extent*	20.0
*rather much*	25.7
*very much*	44.3
Please evaluate your overall experience of the ANZAED credentialing process:	
*extremely poor*	0.9
*poor*	4.0
*fair*	28.2
*good*	52.0
*excellent*	15.0
The ongoing professional development requirements for maintaining your Credential status are reasonable:	
*strongly disagree*	7.8
*somewhat disagree*	7.3
*neither agree nor disagree*	16.0
*somewhat agree*	30.6
*strongly agree*	38.4
The ongoing professional development enhances my understanding of ways to assess and manage eating disorders:	
*strongly disagree*	1.8
*somewhat disagree*	5.4
*neither agree nor disagree*	13.0
*somewhat agree*	36.3
*strongly agree*	43.5
The ongoing professional development increases my confidence to deliver safe and effective treatment to individuals experiencing eating disorders, their families and supports:	
*strongly disagree*	1.8
*somewhat disagree*	4.0
*neither agree nor disagree*	13.5
*somewhat agree*	33.2
*strongly agree*	47.5
The ongoing professional development increases my willingness to provide treatment to individuals experiencing an eating disorder, their families and supports:	
*strongly disagree*	6.9
*somewhat disagree*	4.1
*neither agree nor disagree*	17.5
*somewhat agree*	30.4
*strongly agree*	41.0

### Cross-sectional predictors of clinician’s experience and value of the credential

Linear regression analysis showed that none of the putative explanatory variables (profession, years practising, sector, geographical setting, number of clients with an ED) were significantly associated with clinicians’ overall experience with the Credential. However, the sector in which clinicians worked contributed significant variance to how much they valued the Credential for their individual professional practice (b = -0.903, *p* =.011). Specifically, clinicians working in both public health and private sector (M = 3.38) valued the Credential less than those working in either the public health (M = 4.00), or private sector (M = 4.29) alone.

### Qualitative analysis

**Fig. 1:**
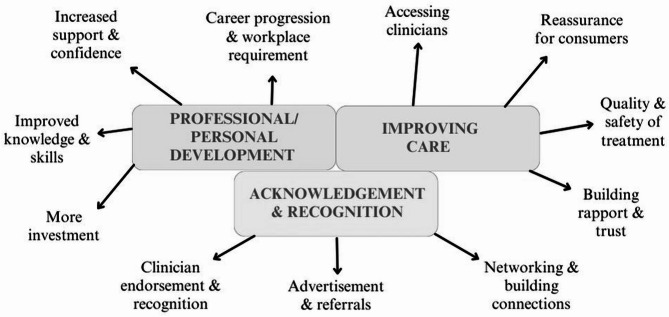
Three broad themes and their subthemes

Content analysis of clinicians’ general views of the Credential and what motivated them to become credentialed generated three broad themes: (i) Professional/personal development; (ii) Improving care; and (iii) Acknowledgement and recognition (Fig. 1). The first theme captured the perceived influence that becoming credentialed had on clinicians’ development both professionally and personally. For example, clinicians often mentioned how becoming credentialed improved their confidence in delivering ED-specific treatment: “[Becoming credentialed is a] *great opportunity for clinicians to access treatment and education to build capacity and confidence in this field of work.”* Participants also talked about how credentialing helped them with upskilling and acquiring new knowledge, which is especially important when working in the ED sphere: *“Credentialing ensures we remain well trained*, *well informed and properly supported with supervision and mentoring.”* Some participants also listed *career opportunities* as one of the benefits of becoming credentialed: *“[The Credential] provides better career growth opportunities for clinicians*.”

The second theme captured the clinicians’ perception of the credentialing system as helping people work within their scope of practice and likely improving care for people living with an ED. Participants mentioned how those seeking treatment had found it easier to locate clinicians and that the Credential helped with standardisation and continuity of care that cultivated trust for those seeking treatment:

*[The Credential] allows a sense of trust to be given to clients that the clinician has a clear understanding and knowledge in the area.*

*Locating eating disorder specific clinicians can present a major barrier to care, delays treatment, and can lead individuals to harmful encounters with healthcare practitioners who are not trained in eating disorder care.*

*For our clients, credentialing provides a reassurance that we are properly qualified by a regulatory body in delivering treatment approaches which are evidence-based.*

The third theme highlighted the clinicians’ feelings of recognition and acknowledgement through the Credential, marking out a distinction between themselves as having expertise in treating EDs. They reported that being credentialed could be used for advertising and thus receiving more referrals as well as connecting with other credentialed clinicians.

*I am grateful for the ongoing recognition this provides of the skills and commitment I hold in this space.*

*[The Credential] allows clinicians to select their specialty and advertise it to potential clients.*

*It’s really important that all clinicians have a minimum level of training, so that clients and GPs can make good referral choices.*

*[The Credential] is a good starting place in building the workforce and supporting identification of other clinicians with a base level of knowledge.*

A few trends emerged when looking into the frequency of codes and themes across participants attributes. Clinicians working in metropolitan areas seemed to have mentioned “accessing clinicians”, “quality & safety of treatment” and “career opportunities” subthemes somewhat more often than those living in rural, regional or remote areas. Moreover, clinicians who worked 1 day or less with ED clients mentioned “increased support & confidence” subtheme more often than those working with ED clients several days per week. When examining profession, psychologists relative to dietitians seemed to have talked more about how being credentialed requires more investment (e.g., time, money). In addition, those working in the private practice sector talked more about advertisement and referrals when compared to those working in public health, or combined public and private.

## Discussion

The vast majority of credentialed clinicians reported that CPD increased their confidence and willingness to deliver ED treatment, and that supervision enhanced their reflective skills and ethical thinking. However, three quarters of clinicians reported that attaining the Credential had not changed the number of ED patients that they were currently treating on a weekly basis. Similarly, in previous research [19] the credentialing system was not well recognised in the early stages of implementation, which is something participants of the present study also highlighted. Nevertheless, it is possible that over time, the ANZAED Eating Disorder Credential would generate more external recognition to facilitate the referral process and thus increase the number of clients with an ED seen by credentialed clinicians. However, this potential benefit is less applicable for credentialed clinicians working in employed capacities via the “public health or other” pathway, for whom personal referrals are not relevant and waiting lists are frequently lengthy. Consequently, in the current study these clinicians less frequently mentioned referrals as one of the benefits of becoming credentialed.

Credentialed clinicians’ views and experience of the Credential were generally positive. Nearly 70% of the participants believed that ongoing professional development requirements were reasonable and that it increased their willingness to provide treatment, whilst 80% reported that ongoing professional development boosted their confidence in delivering treatment. This is in accordance with previous research showing improved confidence and reflective practice following an increase in supervision hours (albeit the sample included only dietitians) [21]. It is likely, that for some clinicians, access to more education as well as being considered a “Credentialed Eating Disorder Clinician” boosted their confidence in addition to receiving support via supervision. Interestingly, those seeing ED clients one day per week or less talked about valuing the increased support and confidence as a result of becoming credentialed to a greater extent relative to those who reported seeing ED clients more often. This suggests that clinicians more frequently involved in ED treatment may feel more confident delivering ED treatment, could already be receiving ED-specific supervision, and perhaps already be connected with other clinicians in the field. These experienced ED clinicians thus may not immediately benefit from the Credential as much as those whose area of practice is not primarily EDs. Furthermore, clinicians working in the private practice sector talked more about advertisement and referrals, and on average seem to value the Credential somewhat higher than those employed in the “public health or other” roles. It seems plausible that those clinicians working in private practices rely more on referrals to sustain their practice and are also able to use the Credentialed Eating Disorder Clinician title to distinguish themselves when promoting their practice, or even in everyday communications (e.g., an email sign-off). Potentially this benefit of becoming credentialed could have been a motivating factor among clinicians working in private practice. Future revisions of the credentialing system may need to consider how to increase endorsement of the Credential among clinicians working in “public health or other” roles. This is somewhat reflected in the finding that Credentialed Eating Disorder Clinicians working in private practice more often mentioned “referrals” and “advertisement” during free text survey responses. In addition, when it came to profession, psychologists more often than dietitians discussed how being credentialed implies more time and money investment, yet clinicians’ profession was not significantly linked to how much they value the Credential.

Another major theme that emerged from the free text survey responses was “improving care” which includes providing reassurance to clients and their families, ensuring easier access to qualified clinicians, and developing trust and rapport with clinicians. Participants mentioned how the Credential helps consumers build trust and reassurance that the care they receive will be adequate, which was shown to facilitate help-seeking [22]. This theme reflects how the Credential can positively affect consumers when it comes to finding appropriately qualified and experienced clinicians (via “find a treatment provider” function), receiving safe and evidence-based treatment, and building strong and lasting therapeutic relationships. The clinicians often mentioned how the Credential ensures treatment safety given that there are some treatment providers who lack ED-specific knowledge and skills and may thus unintentionally cause harm. This highlights the importance of a skilled workforce with the appropriate qualifications, ongoing training, and expert supervision in providing safe and effective ED treatment [14] and the role of the credentialing system in providing a clear set of minimum standards to support the implementation of this [11].

The present study has several strengths such as the use of mixed-methods design, wherein surveys were distributed to allow participants to provide succinct responses and to expand on these responses in more depth via open-ended questions. A robust analysis of qualitative data was achieved by having two authors meet multiple times to review data and identify and reach a consensus, with the help of a third author, on the final thematic map. A large sample size was obtained and was inclusive of each profession eligible for the ANZAED Eating Disorder Credential, and participants were invited directly via email, reducing risk of fraudulent responses. However, one of the limitations of the study is that some professions (e.g. psychiatrists) were under-represented, and the study was conducted prior to the inclusion of a credential specifically for General Practitioners (separate from those who are credentialed as mental health professionals). Future research should aim to explore the perspectives of those General Practitioners who have since become credentialed as well as aim to reach a larger number of credentialed clinicians and to contrast findings with other certification or credentialing programs internationally. Future research may also test hypotheses suggested by the exploratory findings, in particular studying the proportion of patients with an ED cared for by credentialed practitioners over time compared to practitioners with similar levels of training who do not become credentialled. It is important to also evaluate people with EDs perceptions of care from credentialed versus not credentialed clinicians.

Another limitation is that clinicians were asked how becoming credentialed has affected the number of ED clients they see; yet given the recency of the Credential’s formation and the fact that communication and promotion of the Credential to the general public was not widespread, it is unlikely clinicians would have been able to observe this effect. Finally, disproportionately high number of clients with AN diagnosis reported in this study may partially be attributed to the fact that participants were likely more experienced clinicians with a greater case load of people with AN than expected, given the community prevalence.

In conclusion, credentialed clinicians had an overall positive experience with the credentialing system. Clinicians highlighted the role of the Credential in encouraging professional and personal development resulting in improved confidence and willingness for clinicians to treat EDs. The clinicians also valued the Credential as a way of improving care for consumers by emphasising the importance of a skilled workforce, and providing acknowledgement and recognition for clinician’s level of experience, skills, and ongoing commitment to training and supervision in EDs. The credentialing system was valued most by those clinicians who had a smaller case load of ED clients (i.e., equivalent to 1 day or less a week) and by those clinicians who worked in the private practice sector, yet the majority of clinicians had not experienced a change in the number of ED patients they were treating on a weekly basis since attaining the Credential. This suggests that there may be a need to increase the promotion and external recognition of the ANZAED Eating Disorder Credential to ensure visibility for consumers and to support increased referrals for clinicians.

## Data Availability

The datasets used and/or analysed during the current study are not publicly available. They may be available from the corresponding author upon reasonable request and in accordance with Human Research Ethics permissions. Permission for the data to be made publicly available is not sought as it would identify participants.

## Declarations

### Authors’ information

KP is an Adjunct Research Fellow in the School of Medicine at Western Sydney University and a Postdoctoral Research Fellow at the Graduate School of Health at the University of Technology Sydney, JC is an Associate Professor of Clinical Psychology at Western Sydney University, MM is a Research Officer at Western Sydney University, GH is an Adjunct Associate Professor of Medicine at Western Sydney University and Credentialing Director for ANZAED, RB is a Research Officer at Western Sydney University, SM is a Senior Lecturer in the Department of Psychology, Counselling & Therapy at La Trobe University, and PH is a Professor of Medicine at Western Sydney University.

### About this supplement

This article has been published as part of Journal of Eating Disorders Volume 13 Supplement 1, 2025: The Australia & New Zealand Academy for Eating Disorders. The full contents of the supplement are available online at: https://jeatdisord.biomedcentral.com/articles/supplements/volume-13-supplement-1.

## Abbreviations

ANZAED: Australian and New Zealand Academy of Eating Disorders
CPD: Continuing Professional Development
ED: Eating Disorder
GP: General Practitioner
MH: Mental Health
NEDC: National Eating Disorder Collaboration
